# Separation properties and fouling resistance of polyethersulfone membrane modified by fungal chitosan

**DOI:** 10.1186/s13065-024-01341-w

**Published:** 2024-11-11

**Authors:** Hilya N. Iman, Henry Susilo, Adhi Satriyatama, Ignatius D. M. Budi, Kiki A. Kurnia, I. G. Wenten, K. Khoiruddin

**Affiliations:** 1 Department of Chemical Engineering, Faculty of Industrial Technology, Institut Teknologi Bandung, Jalan Ganesa No. 10, Bandung, 40132 Indonesia; 2 Research Center for Nanosciences and Nanotechnology, Institut Teknologi Bandung, Jalan Ganesa No. 10, Bandung, 40132 Indonesia; 3 Biosciences and Biotechnology Research Center, Institut Teknologi Bandung, Jalan Ganesa No. 10, Bandung, 40132 Indonesia

**Keywords:** Fouling, Hydrophilicity, Heavy metal, Wastewater

## Abstract

This research explores the enhancement of polyethersulfone (PES) membranes through the incorporation of chitosan derived from the lignicolous fungus *Ganoderma sp*. Utilizing wet phase inversion and solution casting techniques, chitosan was successfully integrated into the PES matrix, as confirmed by Fourier Transform Infrared Spectroscopy (FT-IR), which indicated a high deacetylation degree of 75.7%. The incorporation of chitosan significantly increased the membrane hydrophilicity, as evidenced by a reduction in the water contact angle and a substantial improvement in pure water permeability, from 17.9 L m^-2^ h^-1^ bar^-1^ to 27.3 L m^-2^ h^-1^ bar^-1^. The membrane anti-fouling properties were also notably enhanced, with the Flux Recovery Ratio (FRR) increasing from approximately 60–80%. Moreover, the chitosan-modified PES/CS membrane, particularly at a 5% chitosan concentration, demonstrated exceptional efficacy in pollutant removal, achieving over 90% elimination of total suspended solids, cadmium (Cd), and lead (Pb), alongside a 79% reduction in color during the treatment of textile wastewater.

## Introduction

Membrane technologies have become increasingly prevalent in water and wastewater treatment, recognized for their efficiency in generating high-quality outputs with minimal chemical interventions. However, the challenge of significant fouling during membrane operations in wastewater treatment persists. Fouling, the accumulation of solutes on or within the membrane, leads to performance decline [1]. In the context of textile wastewater, surfactants and dyes emerge as principal fouling agents [2], with surfactants inducing fouling through hydrophobic interactions [3]. This fouling phenomenon not only diminishes efficiency but also escalates energy and chemical consumption necessary for maintaining output and facilitating membrane cleaning or rejuvenation. Addressing fouling involves a multitude of strategies, such as optimizing pre-treatment processes, operational conditions, membrane modifications, and cleaning protocols [4–7].

Hydrophilization, or the process of enhancing a membrane affinity for water, serves as a strategic measure to prevent the attachment of hydrophobic contaminants. Membranes constructed from hydrophobic materials like polysulfone (PSf) and polyether sulfone (PES) are particularly prone to fouling due to the natural compatibility between the hydrophobic contaminants and membrane material. By increasing hydrophilicity, a membrane can absorb more water, effectively reducing fouling instances [5, 8, 9]. A hydrophilic membrane surface also facilitates the easier removal of foulants, rendering the fouling layer more reversible [10]. Notably, PES membranes, commonly used in ultrafiltration (UF), exhibit inherent hydrophobicity, hence the introduction of hydrophilic agents can significantly mitigate fouling [4, 11, 12].

Chitosan, a naturally occurring biocompatible and biodegradable polymer known for its antimicrobial properties, has been extensively employed to alter membrane characteristics [13–15]. Its hydrophilic nature significantly bolsters membrane resistance to fouling [15, 16]. For instance, Mansourpanah et al. [17] enhanced the surface and antifouling properties of PES membranes through microwave-assisted grafting of chitosan with acrylamide, achieving remarkable antifouling efficacy against bovine serum albumin and enhanced separation capabilities for ion solutions. Furthermore, Afsarian and Mansourpanah [18] introduced a nanofiltration membrane by integrating sodium tripolyphosphate (STPP)-modified chitosan into PES, thus improving salt rejection due to chitosan electric charge. Mousavi et al. [19] developed a thin film nanocomposite membrane by applying a PEBAX/chitosan-coated multiwalled carbon nanotubes layer onto a PES base, demonstrating exceptional antifouling properties, high permeate flow, and over 98% rejection of Malachite green.

Chitosan can also be sourced from fungi [20], offering advantages over crustacean-derived chitosan, such as more uniform physical and chemical characteristics [21, 22]. Fungal chitosan benefits from being unaffected by seasonal shifts or marine pollution and bypasses the need for demineralization [23].

Chitosan can be sourced from both fungi and crustaceans, each offering distinct advantages and characteristics. The core difference between fungal and crustacean-derived chitosan lies in their origins and extraction methods. Crustacean chitosan is obtained from marine exoskeletons through chemical deacetylation, which raises concerns about marine resource depletion and potential allergenic reactions associated with shellfish products [24, 25]. In contrast, fungal chitosan, derived from fungal cell walls, offers a more sustainable alternative, with more uniform physical and chemical properties. It remains unaffected by seasonal shifts or marine pollution and bypasses the need for demineralization [21, 22, 26]. Moreover, fungal chitosan is increasingly valued for its reduced environmental impact and its potential to address resource conservation issues, making it a promising substitute for crustacean-derived chitosan in various applications [23, 27].

Among fungal sources, Ganoderma sp., prevalent in tropical and subtropical forests, presents a viable chitosan source [28]. It is readily cultivable, has medicinal properties, and is considered a safe chitosan source [29–32]. However, the deployment of UF membranes enhanced with fungal chitosan for Batik wastewater filtration and the exploration of related fouling mechanisms remain sparsely documented. This study, therefore, employs chitosan derived from Ganoderma fungus to modify PES membranes, evaluating their performance in real Batik wastewater filtration and analyzing the fouling mechanisms encountered.

## Materials and methods

### Materials

Polyethersulfone (PES) powder, procured from Huaian Ruanke Trade Co., Ltd., China, served as the foundation for the membrane matrix. Wild Ganoderma fungus, sourced from a local supplier in East Java, Indonesia, and commercial polyvinyl pyrrolidone (PVP) (K-90, from a local distributor), functioned as the pore-forming component. Dimethylacetamide (DMAc) (99.9%, acquired from Shanghai Jingsan Jingwei Chemical Co. Ltd.), was utilized as the dissolving agent. *Batik* wastewater, collected from Rumah Batik Lembang in West Java, Indonesia (-6.818213, 107.631669), acted as the membrane feed solution. All experiments employed demineralized water, produced by a lab-scale reverse osmosis unit.

### Chitosan extraction

**Fig. 1:**
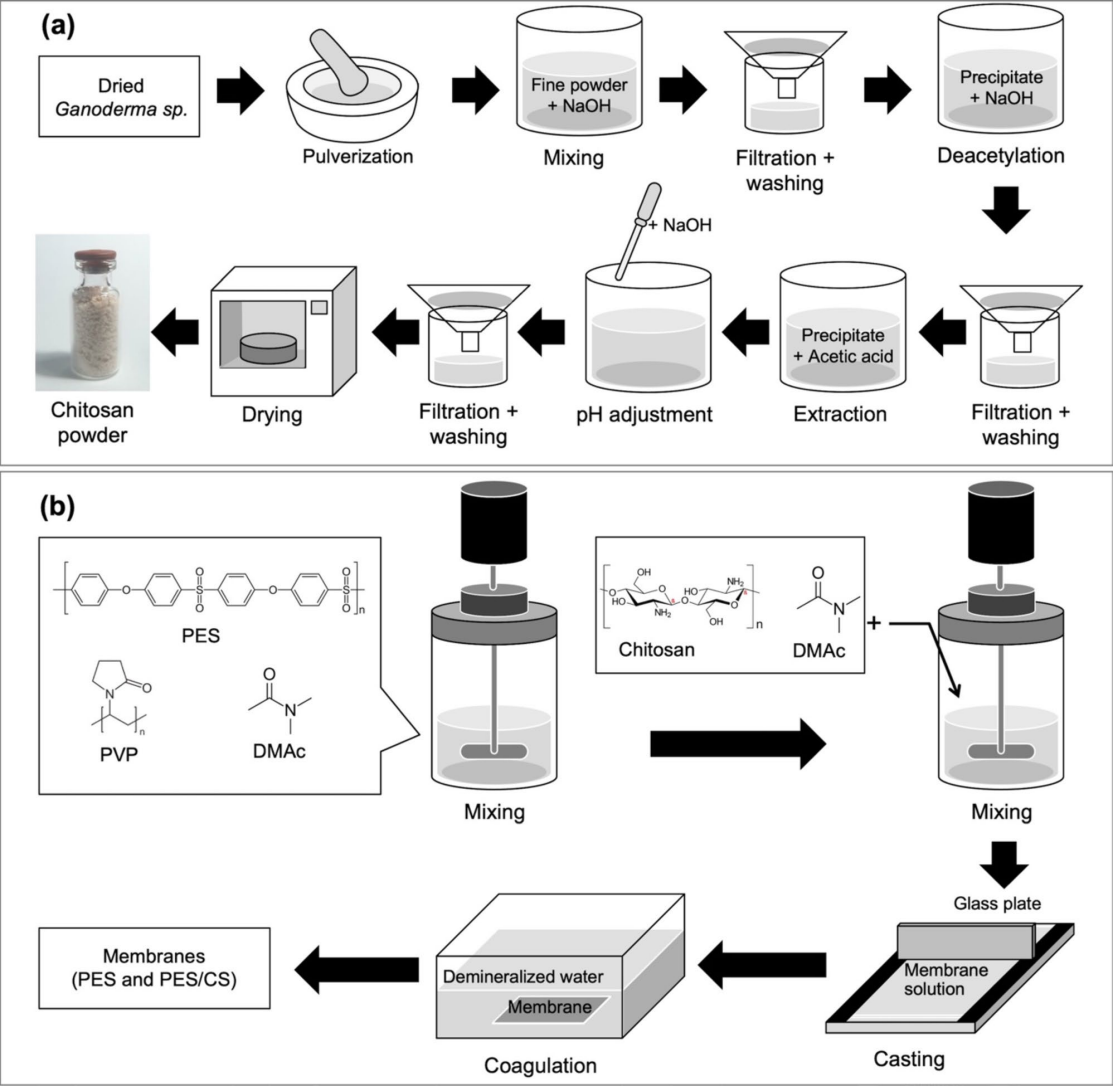
Illustration of (**a**) Chitosan extraction and (**b**) membrane fabrication procedures

The extraction of chitosan from Ganoderma sp. followed documented procedures [33]. The procedure is illustrated in Fig. 1 (a). Initially, the dried Ganoderma sp. mushrooms were finely ground. This powder (3 g) was then dissolved in 1 M NaOH (100 mL) and stirred at 90 °C for 2 h, followed by separation through gravity filtration using filter paper. The suspension was washed with demineralized water until neutral pH was reached. The resulting chitin-containing precipitate underwent alkaline treatment with 40%-w/v NaOH at 100 °C for 2 h for deacetylation. This precipitate was then rinsed with hot demineralized water until neutral pH, extracted with 5% acetic acid in water (30 times the volume) at 90 °C for 3 h, adjusted to pH 10 with NaOH solution, collected, and dried at 60 °C [33].

### PES/CS membrane fabrication

The PES membrane was produced via the wet phase inversion method (Fig. 1b), with the membrane solution composition detailed in Table 1. The process began with dissolving PES and PVP in DMAc, stirred at room temperature for 12 h at 200 rpm [34]. Chitosan, in pre-measured amounts (approximately 0.1 and 0.2 g) dissolved in DMAc (5 mL), was added to the PES solution and stirred at 50 °C for another 12 h. The homogenized solution was cast on a glass plate, allowed to dry at ambient conditions for 5 min, and then submerged in demineralized water for 2 h [16, 35]. The membranes were stored in purified water until further testing. The chitosan concentrations incorporated into the membrane were 2.5% and 5.0% wt. (as detailed in Table 1). These concentrations were chosen because higher concentrations, such as 7.5% and 10%, were found to compromise the membrane structural integrity.

**Table 1:** Membrane solution composition

Membrane code	Ratio of CS/PES	Ratio of PVP/PES	Ratio of (PES + PVP)/DMAc
PES	0:100	9:91	21:79
PES/CS-2.5%	2.5:97.5	9:91	21:79
PES/CS-5.0%	5.0:95.0	9:91	21:79

### FT-IR analysis, acetylation (DA) and deacetylation (DD) degrees, and SEM analysis

Chemical properties of chitosan and membrane were analyzed by Fourier transform infrared spectroscopy (FT-IR, The PerkinElmer SpectrumTwo). The FT-IR spectroscopy was performed at a wavelength of 4000–650 cm^-1^. The degree of acetylation (DA) and deacetylation (DD) of chitosan were determined by the absorbance ratio of A_1655_ to A_3450_ of FT-IR. The formula for calculating DA and DD is as follows [36].

1 $$\:DA\left(\%\right)=\left(\frac{{A}_{1655}}{{A}_{3450}}\right)\times\:100/1.33$$

2 $$\:DD\left(\%\right)=100-DA$$

The membrane morphology was probed by using a scanning electron microscope (SEM, JEOL JSM-6510LA (JEOL Company, Musashino, Tokyo, Japan).

### Pure water permeability

Pure water flux of the membrane was performed by using demineralized water at 1–2 bar trans-membrane pressure (TMP). Pure water flux was calculated using the formula:

3 $$\>{J_w} = {V \over {\Delta t \times \>A}}$$

where $\:{J}_{w}\:$is the pure water flux (L m^-2^ h^-1^ or LMH), *V* is permeate volume (L) collected at time interval t (h), and A is the membrane surface area (m^2^). Pure water permeability was obtained as the slope of pure water flux vs. TMP.

### Wastewater quality analysis

The quality of *Batik* wastewater and membrane permeate were analyzed using procedures according to American Public Health Association (APHA) standards. The parameters of water quality included total suspended solids (TSS) (APHA-2540-D-2012), cadmium (Cd) (APHA-3500-CD-2012), lead (Pb) (APHA-3500-PB-2012), and true color (APHA-2120-C).

Membrane rejection towards pollutants (% rejection) is calculated by:

4 $$\:\text{R}\text{e}\text{j}\text{e}\text{c}\text{t}\text{i}\text{o}\text{n}\:\left(\%\right)=\left(1-\frac{{C}_{p}}{{C}_{f}}\right)\times\:100\%$$

where *C*_*p*_ is the concentration of the component in the permeate and *C*_*f*_ is the concentration of the component in the feed solution.

### Water contact angle

To characterize membrane surface hydrophilicity, water contact angle (WCA) measurement was conducted using a procedure reported in literatures [5, 9]. A drop of demineralized water was placed onto the membrane surface by using a micro-syringe (50 µL; From Shanghai Gaoge Industry and Trade Co. Ltd). Water from about half the volume of the micro-syringe was dropped on the membrane surface. Then, the droplet was photographed, and a contact angle was estimated by ImageJ software.

### Flux recovery ratio (FRR)

First, the pure water flux of the fresh membrane was (*Jw*_*1*_) was measured at a TMP of 3 bar. Then, the membrane was used for *Batik* wastewater filtration for 80 min. The used membrane was backwashed by using demineralized water for 15 min. Afterward, the second pure water flux measurement was carried out (*Jw*_*2*_). FRR is calculated using the following formula:

5 $$\:\text{F}\text{R}\text{R}\left(\text{\%}\right)=\frac{J{w}_{2}}{J{w}_{1}}\times\:100$$

## Results and discussion

### Extract chitosan properties

**Fig. 2:**
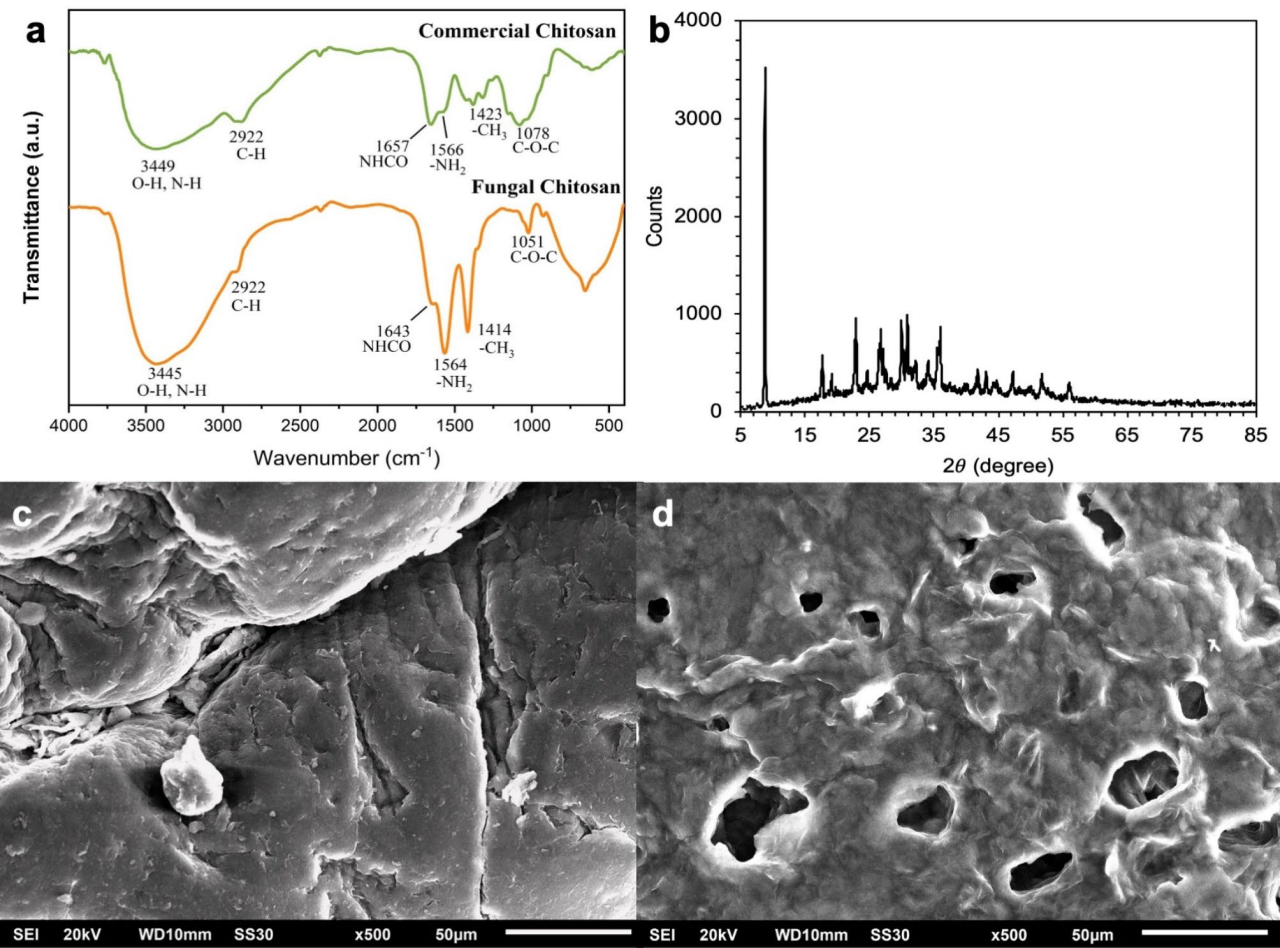
(**a**) FT-IR spectra of commercial chitosan and fungal chitosan extracted from *Ganoderma sp.* (**b**) XRD pattern of fungal chitosan. (**c**) SEM image of extracted chitosan in this work. (**d**) SEM image of commercial chitosan

To enhance the properties of PES membranes with the integration of chitosan, it is crucial to thoroughly characterize the chitosan used. The chitosan derived from fungi, as depicted in Fig. 2(a), was analyzed using Fourier Transform Infrared (FT-IR) spectroscopy. The results were then compared to those of commercially available chitosan from Sigma Aldrich. The analytical results, showcased in Fig. 2(b) and detailed in Table 2, reveal characteristic FT-IR spectral peaks at 1650 cm^-1^, 2900 cm^-1^, and 1030 cm^-1^, attributable to C = O stretch, C-H stretch, and C-O stretch, respectively [40]. Notably, distinctive bands at 1564 cm^-1^ and 1346 cm^-1^, linked to -NH_2_ bending and amide III, respectively, were identified in the chitosan from Ganoderma sp [32]. The amide III band presence corroborates the composition of the extracted chitosan as γ-chitosan, aligning with observations made by Kaya et al. [41]. The commercial chitosan exhibited similar spectral features, validating the effective extraction of chitosan from *Ganoderma sp.* Additional peaks and their associated functional groups are documented in Table 2, consistent with previous studies [40].

**Table 2:** Functional groups and FT-IR peaks of chitosan extracted from *Ganoderma sp.*, commercial chitosan, and chitosan from other work

Group	Chitosan from literature [37]	Chitosan from Literature [38]	Chitosan from Literature [39]	Commercial chitosan	Chitosan extracted from *Ganoderma sp.*
Group tension O-H	3450 cm^− 1^	3441^–1^	3448 cm^− 1^	3449^− 1^	3445 cm^− 1^
Group tension N-H	3292 cm^− 1^	3360^− 1^	3448 cm^− 1^	-	-
Group tension C-H	2919 cm^− 1^ and 2868 cm^− 1^		2883^− 1^	2922 cm^− 1^ and 2881 cm^− 1^	2922 cm^− 1^ and 2909 cm^− 1^
Amide I	1655 cm^− 1^			1657	1643 cm^− 1^
Tension CH_3_	1430 cm^− 1^	942^− 1^		1423	1414 cm^− 1^
Doubling -NH_2_	1550 cm^− 1^		1598^− 1^	1566	1564 cm^− 1^
Amide III	1313 cm^− 1^			1321	1346 cm^− 1^
Antisymmetric tension bridge C-O-C	1154 cm^− 1^	1109 cm^− 1^		1155	-
Tension C-O	1080 cm^− 1^ and 1029 cm^− 1^		1083^− 1^	1078 cm^− 1^ and 1030 cm^− 1^	1051 cm^− 1^ and 1020 cm^− 1^
Anomeric group CH tension	896 cm^− 1^			897^− 1^	927 cm^− 1^

The X-ray Diffraction (XRD) analysis of *Ganoderma sp*.-derived chitosan, presented in Fig. 2(b), demonstrates a distinct and defined XRD pattern with peaks at 2𝜃 = 8.8° and 19.9°, reflecting typical chitosan characteristics [32]. The pronounced peak at 2𝜃 = 8.8°, compared to that at 19.9°, suggests a predominantly amorphous chitosan structure [42]. Crystallinity analysis, performed using Match software, indicated a crystallinity degree of 34.5%, with the remainder being amorphous. This amorphous characteristic is in harmony with prior literature [41], suggesting that the absence of minerals in Ganoderma sp.-derived chitosan contributes to its amorphous nature, offering an advantage over crustacean sources by obviating the demineralization requirement. Moreover, a significant peak around 20.1° in both samples highlights the (001) and (100) planes’ presence in the monoclinic system.

Analysis of the degree of deacetylation (DD) revealed that the chitosan exhibits a DD of 75.7%, within the typical range for commercial chitosan (70–90%) [43, 44], indicating a high prevalence of amide groups. This finding is in accord with Kaya et al. [45], who reported a DD of 73% for chitosan extracted from a medicinal fungus. The extraction process DD can be fine-tuned by varying the conditions, including alkali concentration, duration, and temperature [46]. Nonetheless, it is crucial to manage these parameters judiciously to avoid adversely affecting the chitosan DD [37].

The SEM images (Fig. 2c and d) reveal distinct differences in surface morphology between the chitosan extracted in this study and commercial chitosan. The fungal-derived chitosan exhibits a smoother and denser surface with fewer visible pores, indicating a more uniform structure. In contrast, the commercial chitosan displays a more porous and irregular surface, with larger and more numerous pores. These morphological variations suggest that the extraction method used in this study results in a more homogeneous material, which may influence its performance in applications such as membrane fabrication and adsorption.

### Membrane chemical properties and morphologies

**Fig. 3:**
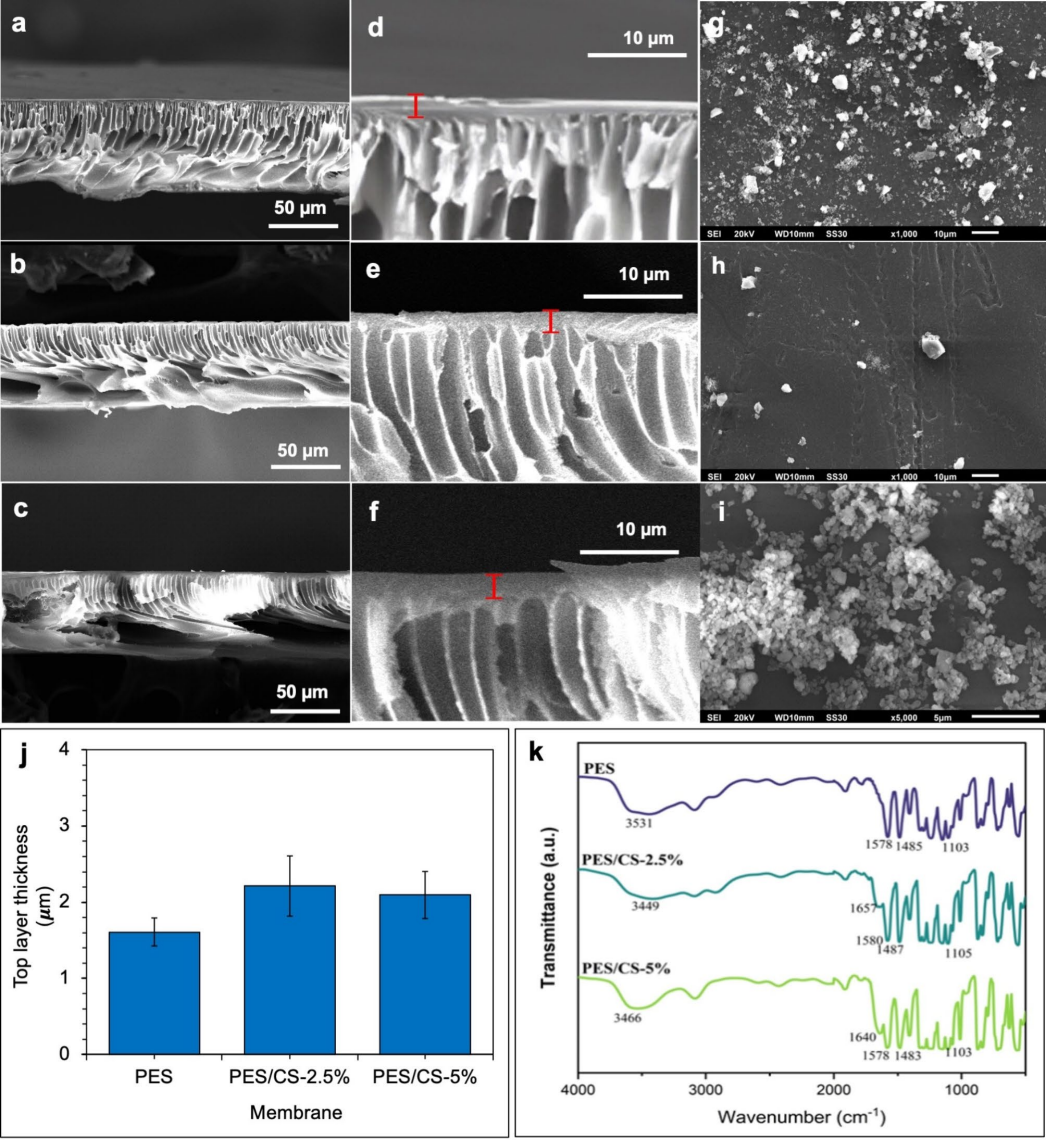
SEM images of (**a**) PES, (**b**) PES/CS-2.5%, and (**c**) PES/CS-5% membranes cross-section. Cross-sections of (**d**) PES, (**e**) PES/CS-2.5%, and (**f**) PES/CS-5% membranes at larger magnification. Top surfaces of (**g**) PES, (**h**) PES/CS-2.5%, and (**i**) PES/CS-5% membranes. (**j**) Top layer thickness of synthesized membranes. (**k**) FT-IR spectra of the membranes

Scanning Electron Microscope (SEM) imagery provided insights into the structural nuances of the PES and PES/CS membranes (Fig. 3a-g). The cross-sectional views highlighted the membranes’ asymmetrical architecture, featuring a dense top layer integral for selective filtration, supported by a porous structure characterized by finger-like projections. The entire membrane showcased a thickness under 100 μm, with the top layer thickness measured around 2 μm, exhibiting standard deviations of 0.2 μm, 0.4 μm, and 0.3 μm for PES, PES/CS-2.5%, and PES/CS-5% membranes, respectively (Fig. 3d-f and h). The observed finger-like and microporous support structure underpins the top layer, ensuring structural integrity and facilitating water transport with minimal resistance. The SEM surface analysis did not indicate a significant variation in the top layer thickness between PES and PES/CS membranes, suggesting that chitosan incorporation does not alter the selective layer thickness. Nonetheless, the membrane pores were too minuscule for surface detection in the SEM imagery. Similar asymmetrical structures with finger-like support and a thin top layer were also noted in PES membranes enhanced by nanocomposites comprising metal-organic frameworks and chitosan [47], where the nanocomposite content modulated the support porosity and top layer thickness, potentially due to variations in membrane formation or solvent-non-solvent exchange dynamics within the coagulation bath. The presence of the metal-organic framework, as reported in [47], significantly influences these outcomes. Furthermore, the degree of deacetylation (DD) of chitosan is known to augment the porosity of PES membranes [48], with higher DD chitosan showcasing increased amine groups, thereby enhancing hydrophilicity [48]. This augmented hydrophilicity, attributable to chitosan inclusion, potentially modulates water molecule interactions with the membrane hydrophilic components during formation.

Surface SEM imagery of the top layer revealed a smooth surface devoid of defects (see Fig. 3g-i), an attribute critical for maintaining membrane selectivity. Membranes enhanced with fungal-derived chitosan, as referenced in [23], demonstrated a more uniform structure compared to those modified with crustacean-sourced chitosan, a variance likely rooted in the different molecular masses of chitosan derived from fungi versus animals [23].

Upon detailed examination of Fig. 3, it is evident that the incorporation of chitosan into the PES membrane matrix significantly influences the morphological and structural properties of the resulting membranes. The SEM cross-sectional images (Fig. 3d and e, and 3f) reveal that the addition of chitosan modifies the architecture of the finger-like pores near the top layer. Specifically, the PES/CS-2.5% and PES/CS-5% membranes (Fig. 3e and f) exhibit more pronounced and elongated finger-like pores compared to the pristine PES membrane (Fig. 3d). This alteration in pore structure can be attributed to the increased hydrophilicity of the casting solution due to the presence of chitosan, which affects the phase separation process during membrane formation [49]. Additionally, the pore density appears to increase with higher chitosan content, with the PES/CS-5% membrane (Fig. 3f) showing a more densely packed array of finger-like pores compared to the PES/CS-2.5% (Fig. 3e) and pure PES membranes (Fig. 3d). This densification is likely to enhance membrane permeability, although it may also impact the mechanical stability, which is critical depending on the application. Furthermore, as depicted in Fig. 3j, the PES/CS-2.5% and PES/CS-5% membranes exhibit a thicker top layer than the unmodified PES membrane. This increased thickness is likely due to the interaction between chitosan and PES, which may lead to a more expansive arrangement of polymer chains within the top layer [50].

The Fourier Transform Infrared (FT-IR) spectroscopy analysis of the PES membranes and those with chitosan content (see Fig. 3k; Table 3) revealed distinct spectral patterns. The spectra of the PES/CS-2.5% and PES/CS-5% membranes exhibited a peak in the 1640–1657 cm-1 region, indicating the presence of an acetylated amine structure, which was absent in the pure PES spectrum. The intensity of this peak increased proportionally with the chitosan concentration, confirming the successful incorporation of chitosan into the membrane matrix. Additionally, both modified and unmodified PES membranes displayed aromatic peaks at 1578 cm^-1^ and 1485 cm^-1^, corresponding to the benzene ring and C-C bond stretching, respectively. A significant peak at 3090 cm^-1^, associated with CH-aromatic stretching, was also identified, a characteristic feature of polyethersulfone [52].

**Table 3:** Comparison of PES and PES/CS membranes

Group	PES Membrane from literature [51]	PES polymer	PES/CS-2.5%	PES/CS-5%
Benzene ring stretching	1580 cm^− 1^	1578^− 1^	1580 cm^− 1^	1578 cm^− 1^
C-C bond stretching	1488 cm^− 1^	1485^− 1^	1487 cm^− 1^	1483 cm^− 1^
Aromatic ether stretching	1244 cm^− 1^	1238^− 1^	1236 cm^− 1^	1233 cm^− 1^
C-O bond stretching	1106 cm^− 1^	1103^− 1^	1105 cm^− 1^	1103 cm^− 1^

### Membrane separation properties

**Fig. 4:**
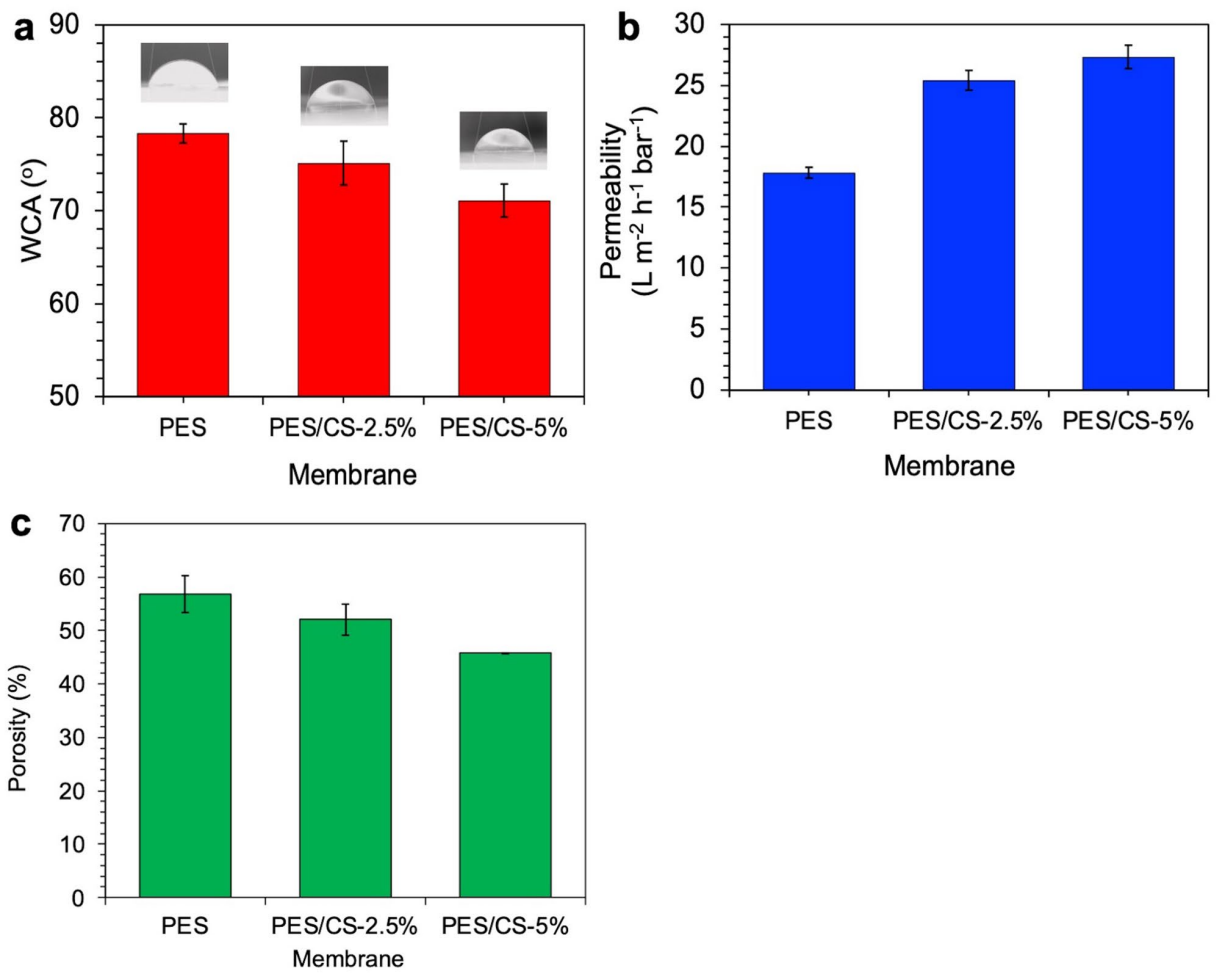
Properties of PES and PES/CS membranes. (**a**) Water contact angle (WCA), (**b**) pure water permeability, and (**c**) porosity

The central aim of integrating chitosan into the membrane formulation was to bolster its hydrophilicity, a critical parameter assessed through Water Contact Angle (WCA) measurements, as illustrated in Fig. 4(a). The baseline PES membrane registered a WCA of 78 ± 1.4°, contrasting with the enhanced hydrophilicity of the chitosan-modified membranes, which recorded WCAs of 75.1 ± 2.4° and 71.1 ± 1.8° for the PES/CS-2.5% and PES/CS-5% variants, respectively. These findings underscore the efficacy of chitosan in elevating membrane hydrophilicity, corroborating similar observations detailed in [15]. This increase in hydrophilicity is attributable to the amide and hydroxyl constituents of chitosan, which are polar functional groups that amplify the water-affinity of the membrane surface. Thus, the incorporation of chitosan not only enhances the membrane hydrophilicity but also potentially augments its anti-fouling capabilities.

Moreover, the hydrophilization process has been observed to improve membrane permeability significantly. As depicted in Fig. 4(b), chitosan addition marked an upsurge in membrane permeability from 18 L m^-2^ h^-1^ bar^-1^ for the unmodified PES membrane to 25 L m^-2^ h^-1^ bar^-1^ and 27 L m^-2^ h^-1^ bar^-1^ for the PES/CS-2.5% and PES/CS-5% membranes, respectively. This enhancement in permeability is directly linked to the membrane increased hydrophilicity, suggesting that chitosan functional groups intensify water-membrane interactions, thus facilitating more efficient water transport through the membrane [23]. The addition of CS to PES membranes introduces functional groups, such as amine and hydroxyl groups, which not only enhance the hydrophilicity but also strengthen hydrogen bonding interactions between the membrane and water molecules, further improving water transport efficiency [53]. Additionally, these functional groups, particularly the amine groups, interact with pollutants through mechanisms such as electrostatic attraction and chelation, especially with metal ions present in wastewater, thereby enhancing the membrane capacity to adsorb and remove contaminants. The interaction mechanism between CS and PES, as well as between the PES-CS membrane and pollutants, aligns with findings in similar systems where the addition of nanoparticles or functional groups to polymer matrices has been shown to improve membrane properties [53].

Conversely, Fig. 4(c) indicates a decline in membrane porosity subsequent to chitosan integration into the matrix, a phenomenon consistent with findings from [54], where chitosan-based membranes typically showcased reduced porosity. This diminished porosity is thought to contribute to the PES/CS membranes’ heightened metal ion rejection capabilities, underscoring the multifaceted impact of chitosan addition on membrane functionality.

### Membrane filtration performance and FRR

**Fig. 5:**
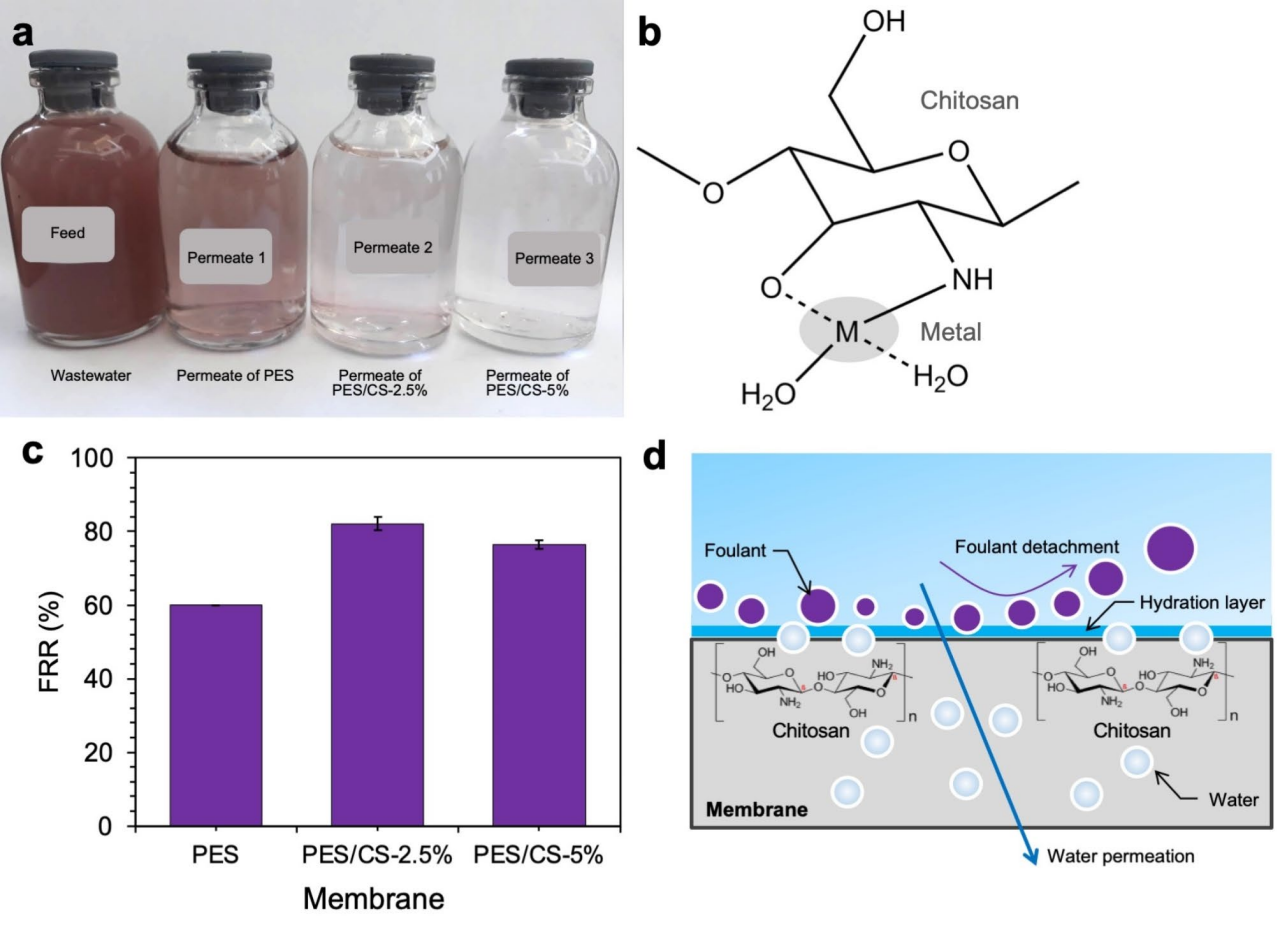
(**a**) Photographs of Batik wastewater (feed) and membrane permeates. (**b**) Possible formation of chelate between chitosan and metals [57]. (**c**) Flux recovery ratio (FRR). (**d**) Schematic illustration of foulant deposition of PES/CS membranes

The developed membranes were applied to filter real wastewater sourced from a Batik manufacturing process, assessing their efficiency in removing specific pollutants, as detailed in Table 4. These tests, performed under a pressure of 2 bars for a duration of 30 min, showed a significant decrease in the concentrations of cadmium (Cd) and lead (Pb) ions, aligning with the American Public Health Association (APHA) standards for wastewater treatment. The chitosan-modified PES membranes demonstrated superior rejection rates for Cd and Pb ions compared to the unmodified PES membrane. Specifically, the PES/CS-2.5% membrane achieved a Cd ion rejection rate of 94.4%, while the PES/CS-5% membrane exhibited an even higher rejection rate of 99.8%. Additionally, Pb ion rejection was 84.8% for the PES/CS-2.5% membrane and exceeded 94.6% for the PES/CS-5% membrane. Moreover, as depicted in Fig. 5a, the color of the wastewater post-treatment showed notable improvements, with the PES/CS-5% membrane achieving a reduction in true color (Pt.Co) by 79.2%.

The enhanced rejection performance of the PES/CS membranes over the standard PES membrane underscores the beneficial impact of chitosan integration on both permeability and pollutant rejection efficiency. This observation is congruent with findings from a prior study [15], which reported improved PES membrane permeability without compromising its ability to reject pollutants through chitosan addition, mirroring the outcomes of this investigation. The heightened removal of metal ions (Cd and Pb) is attributable to the chitosan amino and hydroxyl groups, which can complex with metal ions via chelation mechanisms or electrostatic attractions, particularly under neutral pH conditions [55]. Chitosan inclusion within the membrane matrix augments metal ion binding, thereby elevating removal rates as chitosan content increases. Despite the relatively small size of metal ions, which might allow for their penetration through the membrane selective layer, their interaction with the membrane finger-like structure facilitates their rejection [56]. The chitosan polymer, rich in amine (-NH_2_) and hydroxyl (-OH) groups, provides coordination sites for heavy metal ions, enabling binding through chelation [57] (as illustrated in Fig. 5b) or electrostatic interactions [58].

**Table 4:** Quality of the wastewater and permeate of PES and PES/CS membranes

Parameters	Batik wastewater (ppm)	PES		PES/CS-2.5%		PES/CS-5%	
		Permeate (ppm)	Rejection (%)	Permeate (ppm)	Rejection (%)	Permeate (ppm)	Rejection (%)
Cd (mg/L)​	6.041​	2.714	55.1%	0.341​	94.4%​	0.01	99.8%​
Pb (mg/L)​	0.092​	0.018	80.4%	0.014​	84.8%​	< 0.005​	94.6%​
TSS (mg/L)​	210​0.0	136.0	35.2%	24.0​	88.6%​	8.0​	96.2%​
True color (Pt. Co)	14.3	7.6	47.0%.	2.97	79.2%	1.14	92.0%
COD (mg/L)	57.2	56.5	1.2%	51.6	9.8%	42.2	26.2%
BOD (mg/L)	20.9	20.8	0.4%	18.1	13.4%	14.8	29.2%

In wastewater filtration, fouling poses a significant challenge, particularly when dealing with Batik wastewater, which is characterized by organic compounds (BOD 20.9 mg/L, COD 57.2 mg/L) and solids (TSS 210 mg/L). These constituents have the potential to adhere to the membrane surface, creating a fouling layer that impedes filtration efficiency. In this study, both PES and chitosan-modified PES (PES/CS) membranes were evaluated for their fouling resistance, measured through their Flux Recovery Ratio (FRR) after undergoing backwashing with clean water post-filtration. The FRR outcomes for the PES and PES/CS membranes are illustrated in Fig. 5c.

The results, as depicted in Fig. 5c, highlight that PES/CS membranes achieve higher FRR values compared to the PES membrane alone, suggesting that the incorporation of chitosan significantly enhances fouling resistance. This enhancement in FRR with chitosan addition aligns with previous studies [59], indicating chitosan efficacy in fouling mitigation. Specifically, the FRR for the PES membrane was observed to increase to 82% upon the addition of 2.5% chitosan. This improvement can be attributed to chitosan capacity to attract water molecules, forming a hydration layer on the membrane surface [51, 60]. This hydration layer is instrumental in deterring foulant adsorption and diminishing the fouling rate, as illustrated in Fig. 5d.

It is noteworthy, however, that the FRR value experiences a slight reduction to 76.4% with the incorporation of 5% chitosan. Although this decrease in FRR is relatively minor (under 10%), it could be deemed negligible or within the experimental error margin. In essence, the integration of chitosan into membrane formulations not only shows promise in boosting separation performance but also in enhancing the membrane resistance to fouling, thereby underscoring the beneficial impact of chitosan on membrane technology in wastewater treatment applications.

### Fouling mechanism

**Fig. 6:**
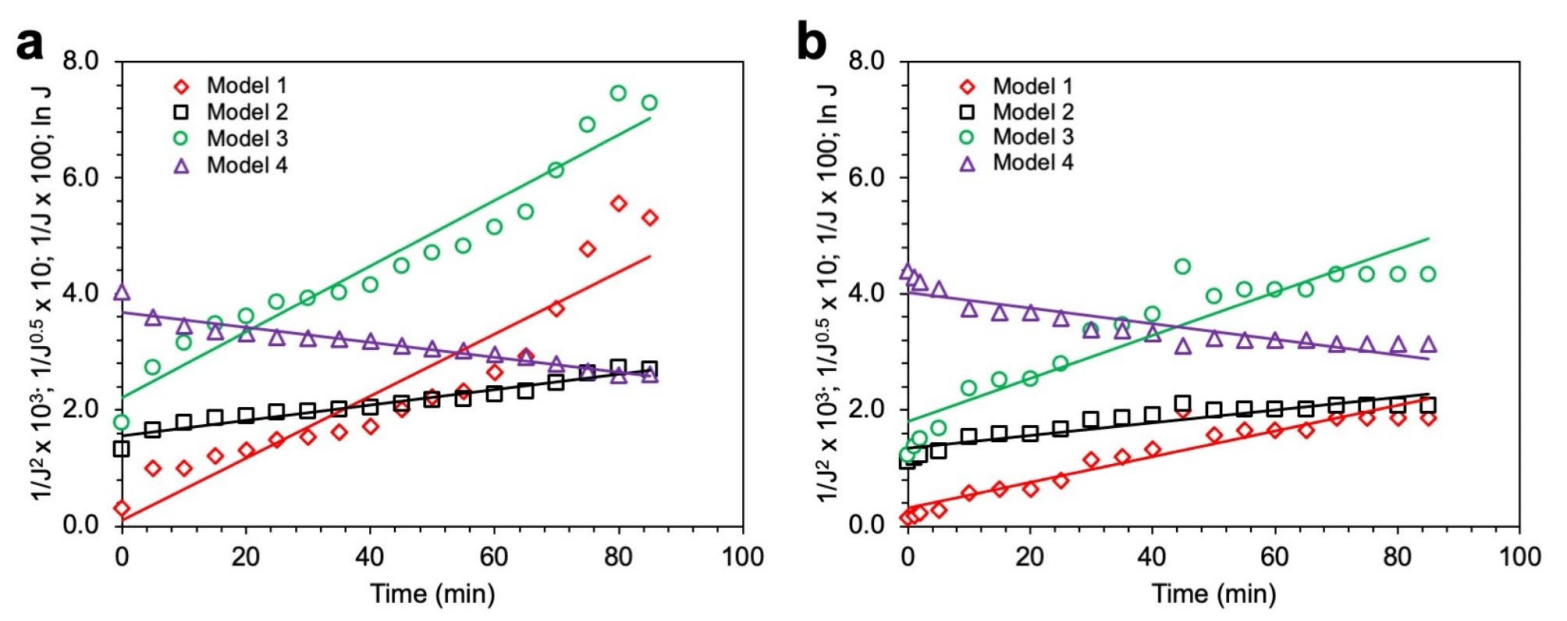
Flux of membrane fitted with Hermia’s model. (**a**) PES membrane. (**b**) PES/CS-5% membrane

**Fig. 7:**
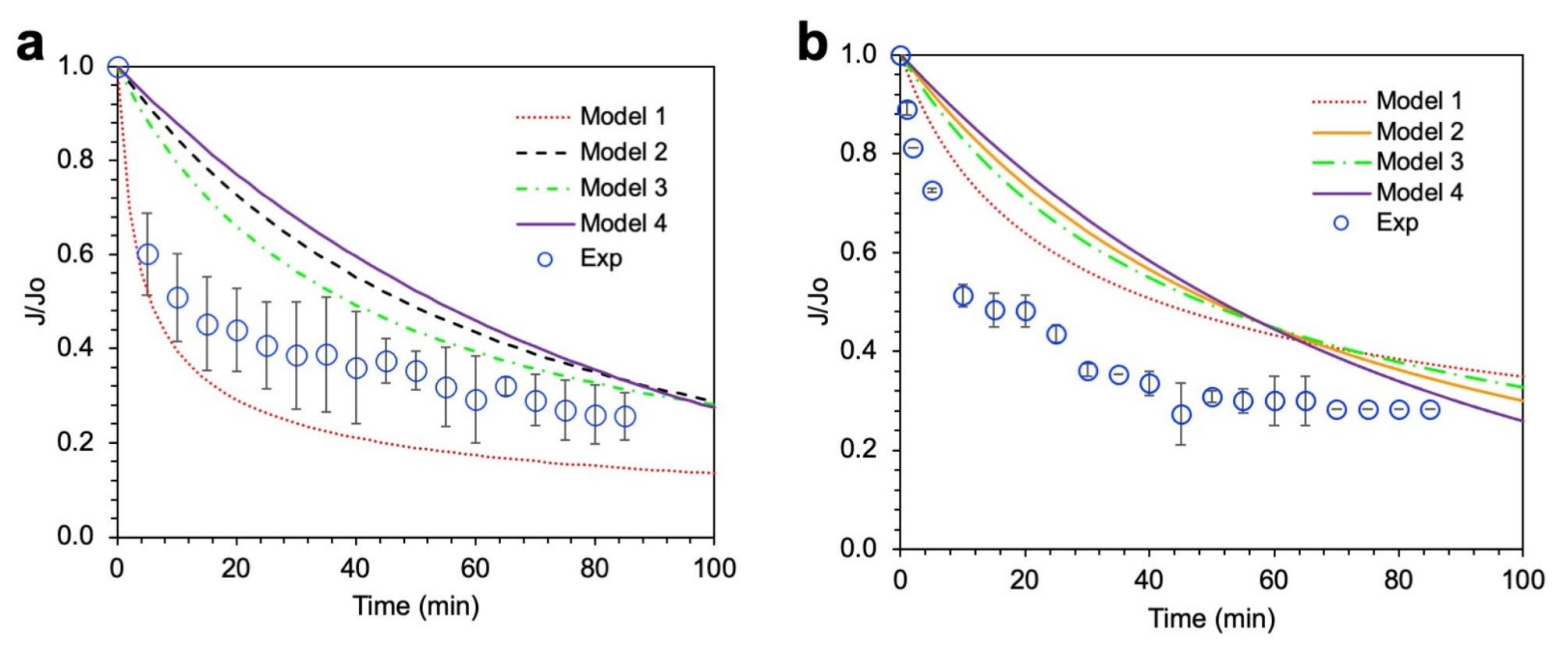
Membrane flux simulated by using Hermia’s model for (**a**) PES and (**b**) PES/CS-5% membranes

The investigation into the fouling mechanisms of synthesized membranes during textile wastewater filtration employed Hermia’s models [61], offering a structured approach to decipher fouling dynamics in dead-end membrane filtration processes. A synopsis of these models is encapsulated in Table 5. The flux profiles of both PES and chitosan-modified PES (PES/CS) membranes, analyzed against Hermia’s models, are showcased in Fig. 6a and b, with the corresponding R^2^ values for each model detailed in Table 5. Following this, the normalized flux was modeled for each scenario, with findings illustrated in Fig. 7a and b.

The analysis, rooted in the R^2^ values, suggests that fouling on the PES membrane primarily manifests through standard and intermediate blocking. Conversely, cake layer formation is identified as the prevailing fouling mechanism on the PES/CS membrane, as evidenced by the highest R^2^ correlation. Cake layer formation, characterized by solute accumulation on the membrane surface, exacerbates resistance to water flow due to mass transfer hindrances [62], a phenomenon corroborated by solute rejection metrics in Table 3. The potential for varied solutes to deposit within membrane pores, prompting standard and intermediate blocking, arises particularly in instances of diminished rejection rates. The analysis further posits that diverse fouling mechanisms might concurrently transpire, attributable to the multifaceted nature of foulants in the feed water, as supported by simulation outcomes in Fig. 7a and b.

Moreover, Table 5 delineates a reduced fouling layer thickness for the PES/CS membrane relative to its unmodified counterpart, underscoring a more favorable fouling parameter profile for the chitosan-enhanced membrane. Notably, a diminished fouling parameter (K) value signals a less substantial fouling layer [63], reinforcing the notion that chitosan significantly augments the membrane resistance against fouling. These insights not only illuminate the differential impact of chitosan modification on fouling dynamics but also affirm the role of chitosan in bolstering the antifouling prowess of membranes in textile wastewater treatment applications.

**Table 5:** Fouling mechanisms, model, and fouling parameters obtained from Hermia’s model

Fouling mechanims	Model	Equation	*R* ^2^		Fouling parameter (K)	
			PES	PES/CS-5%	PES	PES/CS-5%
Cake formation	Model 1	*1/J = 1/Jo*^*2*^ *+ K*_*cf.*_*t*	0.8677	0.8993	5.35 × 10^− 5^	2.22 × 10^− 5^
Standard blocking	Model 2	*1/J* ^*0.5*^ *= 1/Jo* ^*0.5*^ *+ K*_*sb*_ *t*	0.9425	0.8378	1.34 × 10^− 3^	1.11 × 10^− 3^
Intermediate blocking	Model 3	*1/J = 1/Jo + K*_*ib*_ *t*	0.9400	0.8686	5.67 × 10^− 4^	3.71 × 10^− 4^
Complete blocking	Model 4	*ln (J) = ln (Jo) – K*_*cb*_ *t*	0.9133	0.7965	1.30 × 10^− 2^	1.35 × 10^− 2^

## Conclusion

This research successfully extracted chitosan from the wood fungus *Ganoderma sp.* and utilized it to enhance PES membranes. FT-IR spectroscopy confirmed the chemical integrity of the chitosan obtained, with a degree of deacetylation (DD) at 75.7%, aligning with commercial chitosan standards. The integration of chitosan into the PES membrane using a simple blending technique was validated through FT-IR analysis, indicating effective incorporation.

The modification with chitosan significantly enhanced the membrane hydrophilicity, as indicated by the reduced water contact angle. This improvement is attributed to the amide and hydroxyl groups in chitosan, which also led to a substantial increase in pure water permeability—from 17.9 L m^-2^ h^-1^ bar^-1^ to 27.3 L m^-2^ h^-1^ bar^-1^. Moreover, the incorporation of 2.5% chitosan notably enhanced the FRR, elevating it from around 60% to approximately 80%. Fouling analysis further revealed a decrease in fouling resistance for the chitosan-modified membrane, suggesting chitosan efficacy in mitigating irreversible fouling and promoting easier fouling removal via backwashing.

In terms of wastewater treatment efficacy, the modified PES/CS membrane showed remarkable performance in removing cadmium, lead, and TSS, with over 90% efficiency for the PES/CS-5% membrane. The superior metal removal capability is primarily ascribed to chitosan adsorptive properties, rather than to membrane porosity changes. This is due to chitosan multiple chelation sites and its amino and hydroxyl groups, which form strong bonds with metal ions through coordination or ion exchange mechanisms [64–66].

These results underscore the PES/CS membrane potential for Batik and other dye wastewater treatments, highlighting chitosan role in enhancing membrane performance and fouling resistance. Nonetheless, future work should focus on evaluating the membrane longevity and the stability of chitosan within the membrane structure under prolonged operational conditions.

## Data Availability

Data available upon request.

## Abbreviations

APHA: American Public Health Association
CS: Chitosan
DA: Degree of Acetylation
DD: Degree of Deacetylation
DMAc: Dimethylacetamide
FRR: Flux Recovery Ratio
FT-IR: Fourier Transform Infrared Spectroscopy
PES: Polyether Sulfone
PVP: Polyvinyl Pyrrolidone
SEM: Scanning Electron Microscope
TMP: Transmembrane Pressure
WCA: Water Contact Angle
